# *Leaving A Mark*, An Animal-Assisted Intervention Programme for Children Who Have Been Exposed to Gender-Based Violence: A Pilot Study

**DOI:** 10.3390/ijerph16214084

**Published:** 2019-10-24

**Authors:** Alexander Muela, Josune Azpiroz, Noelia Calzada, Goretti Soroa, Aitor Aritzeta

**Affiliations:** 1 Department of Personality, Evaluation and Psychological Treatment, University of the Basque Country UPV/EHU, 20018 San Sebastián, Gipuzkoa, Spain; goretti.soroa@ehu.eus; 2 Biak Bat Association, 31800 Alsasua, Spain; psicologia@biakbat.eus (J.A.); info@biakbat.eus (N.C.); 3 Department of Basic Psychological Processes and Development, University of the Basque Country UPV/EHU, 20018 San Sebastián, Gipuzkoa, Spain; aitor.aritzeta@ehu.eus

**Keywords:** Gender-based violence, Intimate partner violence, Domestic violence, Animal-assisted intervention, Animal-assisted therapy

## Abstract

Gender-based violence is one of the most serious social and health problems faced by women around the world. Importantly, it has a negative impact not only on the woman’s physical and mental health, but also on all members of the family system in which it takes place. The aims of this study were to implement *Leaving a Mark*, an animal-assisted intervention (AAI) programme for children who have been exposed to gender-based violence, and to examine its effect on their associated clinical symptoms. The participants were 19 children (13 boys and 6 girls; *M*_age_ = 8.89, *SD* = 2.23) who had been exposed to domestic violence perpetrated either by their father or their mother’s intimate partner. Clinical symptoms were assessed using the Child Behaviour Checklist (CBCL). After taking part in the AAI programme, the children showed a reduction in internalizing symptoms and in symptoms associated with post-traumatic stress disorder. However, no significant changes were observed in externalizing symptoms or in affective and behavioural dysregulation (CBCL-Dysregulation Profile). These results provide preliminary support for the use of the *Leaving a Mark* programme with children who have been exposed to domestic violence. However, further studies with a larger sample and more rigorous design are required.

## 1. Introduction

The United Nations defines violence against women as “any act of gender-based violence that results in, or is likely to result in, physical, sexual or psychological harm or suffering to women, including threats of such acts, coercion or arbitrary deprivation of liberty, whether occurring in public or in private life” [1] (Article 1 of the Declaration on the Elimination of Violence Against Women). Violence of this kind is one of the most serious social and health problems faced by women around the world. Indeed, the World Health Organization [2] considers violence against women to be a global health problem of epidemic proportions, with about 1 in 3 women (35%) having experienced physical and/or sexual violence in their lifetime.

Most cases of violence against women occur in the home and/or in the context of an intimate partner relationship. Importantly, its consequences are often fatal: official data [2] indicate that between 38% and 50% of murders of women are committed by intimate partners, usually men.

Numerous studies [3,4,5,6] have also highlighted the serious impact that gender-based violence has on women’s physical and mental health. A high percentage of victims suffer some kind of physical injury (musculoskeletal, genital trauma, etc.), while in the area of sexual and reproductive health, the possible repercussions include unwanted pregnancies, abortion, gynecological problems, and sexually-transmitted diseases [7]. Serious problems have likewise been observed in relation to perinatal/maternal health, notably premature deliveries, low birth weight babies, and pregnancy loss [2,8]. Victims of intimate partner violence also tend to have poorer general health (hypertension, cardiovascular problems) [9].

Intimate partner violence is also an important risk factor for emotional and behavioural problems [10], including anxiety and depression, post-traumatic stress disorder, eating disorders, substance abuse, and sleep disorders [5,11]. In addition, victims are more likely to experience suicidal ideation and attempt suicide [12]. The serious impact which the experience of violence has on women also means that they are at a higher risk of disability or early death [5].

Importantly, gender-based violence has a negative impact not only on the woman’s physical and mental health, but also on all members of the family system in which it takes place [13]. In this context, UNICEF [14] reports that 1 in 4 (176 million) children under age 5 live with a mother who has been a victim of intimate partner violence. Retrospective surveys of adults also suggest high prevalence rates of exposure to gender-based violence during childhood and adolescence [15].

Preventing gender-based violence and promoting therapeutic initiatives for children who are exposed to it are priority objectives of the United Nations’ *Global Strategy for Women’s, Children’s and Adolescents’ Health (2016-2030)* [16]. However, for many years, and in many countries, the children of women who were subject to this kind of violence were invisible victims of this social and family problem, and consequently they received neither recognition nor the necessary psychosocial support [17].

There are now various psychological treatments aimed at helping children and young people recover from traumatic experiences and achieve greater psychological wellbeing [18]. Nevertheless, given the enormous social and economic costs that gender-based violence has for families, communities, and society as a whole [2], there is a need to identify and promote effective therapeutic initiatives for children who have been exposed to it.

### 1.1. Impact of Gender-Based Violence on Children

Several systematic reviews [13,19] and meta-analyses [20,21,22,23] have indicated that exposure to gender-based violence can seriously undermine the healthy development of children and adolescents. Moreover, the negative impact appears to be independent of the child’s age at the time [20] and degree of exposure, that is, the consequences are serious, regardless of whether children have merely witnessed domestic violence, been involved in it, or have themselves been a victim of verbal or physical aggression [24].

Exposure to domestic violence has been associated with both internalizing and externalizing symptoms in children and adolescents, since these young people often present symptoms of anxiety and/or depression, alongside aggression or other problem behaviours [20,21,22]. They may also experience symptoms associated with post-traumatic stress disorder, such as recurrent involuntary and intrusive distressing memories of the traumatic event, anxiety dreams, and heightened arousal and reactivity [20]. Research also suggests that child witnesses of domestic violence have greater social and academic difficulties than their peers who have not had such an experience [21]. In this context, it should be noted that individuals exposed to gender-based violence in childhood tend to have problems with psychosocial adjustment subsequently during adolescence and adulthood [25,26].

Regarding gender differences, research suggest that boys and girls are affected in different ways by exposure to domestic violence. Generally speaking, boys tend to show more externalizing behaviours [20,23], whereas girls present more internalizing symptoms [13], although this latter difference has not been confirmed by meta-analytic studies [20,23].

### 1.2. Animal-Assisted Intervention with Children who Have Been Exposed to Gender-Based Violence

Animal-assisted interventions (AAIs) are structured activities involving animals that are employed in educational or health settings with the aim of achieving certain therapeutic benefits or improvements in health and wellbeing [27]. The use of AAIs is an emerging field within the broader context of psychosocial intervention [28,29], notably in the treatment of children and adolescents who have experienced childhood trauma. A key premise here is that the innate qualities of animals can facilitate and maximize the benefits of the therapeutic process [30]. For example, research has shown that interacting with or being in the presence of animals can reduce symptoms of anxiety and depression [31] and improve certain physiological markers of the stress response and wellbeing [32]. More specifically, it has been associated with a lowering of blood pressure [33], a reduction in levels of cortisol [32], lower levels of neurohormones (epinephrine and norepinephrine) and decreased cardiopulmonary pressure [34], and the promotion of increased oxytocin secretion [35]. Studies also suggest that the inclusion of animals can boost treatment adherence and motivation [36], which is a key aspect to consider in the context of therapy for individuals who have suffered traumatic experiences, among whom dropout rates tend to be high [37].

Importantly, a number of recent studies have reported promising results when using an AAI with the aim of improving health and preventing risk behaviours among children and adolescents who have suffered traumatic experiences [38,39]. In general, the use of such interventions has been associated with an improvement in symptoms of post-traumatic stress, depression, and anxiety [36]. Research also suggests that AAIs may help to improve psychosocial adaptation and resilience [30,40] and promote more secure attachments [41]. The animal most commonly used in these interventions is the dog, although some programmes have exclusively involved horses, cats, dolphins, or farm animals. It should be noted that no substantial differences in treatment effects have been observed in relation to the kind of animal used [28,29,38].

To our knowledge, however, there are no treatment protocols designed specifically for children and adolescents who have been exposed to gender-based violence, or studies that have examined the effect of AAIs exclusively in this population.

### 1.3. The Present Study

In light of the above, our primary aim here was to study the effects of a pilot AAI programme on the clinical symptoms (internalizing and externalizing symptoms and symptoms associated with post-traumatic stress disorder) of a group of children who had been exposed to domestic violence. Our expectation was that these children would present fewer symptoms following their participation in the AAI programme.

A more specific objective was to examine whether there were differences in clinical symptoms between boys and girls, both before and after the treatment programme. Our hypothesis here was that boys would present more clinical symptoms, especially of an externalizing kind.

Finally, an issue that has been studied in children and adolescents with mental disorders or symptoms of post-traumatic stress disorder [42,43], but which has not been sufficiently explored in children exposed to domestic violence is the presence of severe affective and behavioural dysregulation based on the Child Behaviour Checklist (CBCL-Dysregulation Profile, CBCL-DP). It has been found that children with elevated scores on the CBCL-DP tend to be at risk of juvenile bipolar disorder, post-traumatic stress disorder, or attention-deficit hyperactivity disorder [44]. A high CBCL-DP score has also been associated with suicidality [45,46] and comorbidity with other behavioural disorders [47]. Importantly, elevated CBCL-DP scores in childhood have been linked to severe psychiatric problems in adolescence and adulthood [46,48,49]. Our expectation here was that children’s scores on the CBCL-DP would decrease following their participation in the AAI programme.

## 2. Materials and Methods

### 2.1. Participants

The sample comprised of 19 children (13 boys and 6 girls) aged between 6 and 15 years (*M* = 8.89, *SD* = 2.23) from the region of Navarre in north-east Spain. During the year prior to their inclusion in the study, all the children had been exposed to domestic violence perpetrated against their mother, either by their father or their mother’s intimate partner (all men).

At the time of the study, all but one of the mother-child dyads lived apart from the perpetrator of the violence and they were all in contact with social services (either the domestic violence or the child protection team), through which they could receive legal advice and psychological support. All the cases included in this study were recruited through these social services teams.

In those cases where the perpetrator was the biological father, 68% of children continued to have regular contact with him, either face-to-face or by telephone. In the remaining 32% of cases, all contact with the father had been severed.

Potential participants were excluded if they had a recognized conduct disorder or a history of cruelty to animals that might prevent them from interacting positively during the AAI programme.

Finally, it should be noted that all the mothers had either received psychological care prior to the intervention or did so during the study period.

### 2.2. Instruments

Child Behaviour Checklist 6–18 (CBCL 6–18) [50]: The CBCL is used to assess behavioural and emotional problems in children and also includes competence scales for activities, social relations, and school. Demographic information and information about possible illnesses/disabilities, concerns about the child, and ratings of positive behaviour are also recorded. The Spanish version of the CBCL that was used in this study contains 113 items that are rated by the parent or primary caregiver on a three-point Likert-type scale (0 = not true (as far as you know), 1 = somewhat or sometimes true, and 2 = very true or often true). The timeframe for item responses is the past six months. Examples of item statements include “argues a lot” and “sleeps less than most kids”. The questionnaire takes approximately 20–30 minutes to complete.

The CBCL comprises of the following eight syndrome scales: Anxious/Depressed, Withdrawn/Depressed, Somatic Complaints, Social Problems, Thought Problems, Attention Problems, Rule-Breaking Behaviour, and Aggressive Behaviour. These can then be combined into two broad band scales—Internalizing Problems (sums the ratings for the Anxious/Depressed, Withdrawn/Depressed and Somatic Complaints scales) and Externalizing Problems (sums the ratings for the Rule-Breaking Behaviour and Aggressive Behaviour scales)—and also yield a Total Problems score (sum of ratings for all the problem items). In addition, item responses are used to provide ratings on the following DSM-oriented scales consistent with DSM 5 diagnoses: Affective Problems, Anxiety Problems, Somatic Problems, Attention Deficit Hyperactivity Disorder, Oppositional Defiant Problems and Conduct Problems.

The CBCL can also be used to assess symptoms associated with post-traumatic stress disorder (the CBCL-Post-Traumatic Stress Problems Scale, CBCL-PTSP) and affective and behavioural dysregulation (CBCL-Dysregulation Profile, CBCL-DP). The CBCL-PTSP is a modified version of scales used by Wolfe and colleagues [51]. In the present study, we used the 14 CBCL items described by Ayer et al. [44].

The CBCL-DP was calculated by summing scores for items on the Anxious/Depressed, Attention Problems and Aggressive Behaviour syndrome scales, a total of 41 CBCL items (maximum score = 82).

The validity of the CBCL is supported by numerous studies conducted in various societies and cultures [52,53], and the instrument shows satisfactory psychometric properties for the Spanish population (Cronbach’s alpha above .80) [54]. In the present study, the scales used showed adequate internal consistency (Cronbach’s alpha of .92, .89, .85 and .92 for Internalizing Problems, Externalizing Problems, the CBCL-PTSP, and the CBCL-DP, respectively).

### 2.3. Procedure

The study involved three stages. We began by contacting the social services’ domestic violence and child protection teams in the region where the study was conducted, explaining the nature of the study and requesting their help in recruiting participants. These teams then put us in touch with mother-child dyads who were interested in taking part. In all cases, we obtained informed consent from the mother, as well as verbal consent from the child/adolescent, before beginning any data collection. We then proceeded to record sociodemographic information and check that potential participants fulfilled the inclusion criteria.

In the second stage of the study, the mothers were asked to complete the CBCL, and subsequently we began to implement the AAI programme. The interventions took place in 2017, 2018, and 2019. A total of four dogs (three Labradors and one Golden Retriever) were used as therapy animals in the interventions corresponding to the present study. Each one was trained as a therapy dog by a specialist trainer using positive reinforcement techniques. It should be noted that all necessary measures were taken throughout to safeguard the welfare of the animals. To ensure the well-being of the participants, all the dogs were subject to prophylactic veterinary treatments prior to the AAI programme (vaccinations and external and internal de-worming to avoid zoonotic risks). The professional team responsible for implementing the programme comprised of two psychologists, both with extensive experience in mental health and psychotherapy.

The AAI programme, *Leaving a Mark*, comprises of five modules (establishing a secure base, psychoeducation, arousal regulation and stress management, emotion expression and regulation, and conclusion) and a total of 14 weekly sessions, each lasting one hour (see Table 1). In developing and planning this programme, we drew upon the work of Rizo et al. [18] and the principles of two psychotherapeutic models: trauma-focused cognitive-behavioural therapy [55] and animal-assisted intervention for victims of childhood trauma [30].

The aims of the first module are to create a therapeutic setting, build a positive therapeutic alliance, and establish a secure base from which to explore the child’s experiences of domestic violence. Thus, the psychologist begins by addressing with the child issues such as the frequency and duration of sessions, the work that will be done both in and between sessions, and confidentiality. The child’s expectations regarding the treatment are also explored. In addition, the child gets to meet the therapy dog and experience for the first time what an AAI entails. Specifically, the child is given the opportunity to play with the dog and carry out tasks such as grooming and feeding, always in accordance with what he or she wishes to do and feels comfortable with.

The second module comprises of two individual sessions and one joint session in which the child’s mother is also present. The primary aim of this psychoeducation module is to normalize the child’s and the mother’s response to the traumatic event and to foster an accurate and non-judgmental account of their experiences. Thus, general information is shared with both mother and child about a number of issues: domestic violence and its consequences, the number of children who are exposed to such events, the need for social institutions to protect women and children against domestic violence and the role of society in ensuring their wellbeing, and the reasons why many children do not talk about their experiences and feelings in this respect. More scientific information about common emotional and behavioural responses to this kind of violence is also provided. The purpose here is to support both mother and child emotionally and to help them see their response to the violence as an adaptive, survival-related response. On a practical level, this work is done by means of a children’s story book adapted to different ages (6–7 years, 8–11 years, and 12–16 years), interspersed with activities involving the therapy dog.

The module related to arousal regulation and stress management involves one individual session and one joint session with the mother. The main objective here is to help the child achieve a permanent reduction in his or her state of arousal, which is invariably high, following exposure to domestic violence. The child is first shown techniques of relaxation [56] and stress management in an attempt to help him or her recover more pleasant bodily sensations. Following each part of the exercise, the child interacts with the therapy dog, which acts as reinforcement and support. In order to help the child become more aware of his or her state of arousal, he or she is asked to imagine a traffic light, both before and after the relaxation exercises. The purpose of the joint session is to introduce these techniques to the mother so that she can help her child practice them at home.

The fourth module focuses on emotion expression and regulation, and it is the longest of the five (comprising four individual sessions and one joint session). The reason for this greater emphasis is that children who have experienced traumatic events are generally overwhelmed by the intensity of their feelings, and they do not normally have the skills required to describe, modulate, tolerate, and overcome them [55]. Thus, this module aims to help them identify and recognize different emotions, to encourage them to express their feelings, and to improve in general their emotion expression and regulation skills. This is achieved through various exercises involving the therapy dog. For example, the child is asked to write down on a piece of paper all the emotions that he or she knows; if their list is very short, they are helped to think of more. Five basic emotions are then chosen from the list and matched to a colour (e.g. happy: yellow; sad: blue, angry: red; frightened/scared: black; calm: green). Next, five balls of the same colours are placed on the floor and the therapy dog is told to bring them over, one at a time. The task for the child is to identify the emotion associated with the colour of the ball, and then to describe a situation in which he or she has felt like that.

The final module is designed to bring the intervention to a close, providing the opportunity for review, and it involves one individual session and one joint session with the mother. It should be noted that the *Leaving a Mark* programme includes homework tasks that provide an opportunity for the child to practice the skills that have been learned in the sessions. Furthermore, throughout the programme, each child has his or her own individual workbook in which to do the written exercises set during the sessions (e.g. a child may be asked to draw a sad child or to describe an experience associated with this emotion), to write down the homework tasks that need to be done and to make a note of any personal impressions and of the commitments made as part of the therapeutic contract (e.g. agreeing to do the homework tasks). At the end of each session, the workbook is kept and stored confidentially by the therapist, but at the end of the intervention programme it is given to the child to keep.

In the third and final stage of the research, all the mothers were asked to complete the post-test measure (the CBCL) two weeks after finishing the treatment programme.

The study was approved by the Ethical Review Board of the University of the Basque Country (Spain).

## 3. Results

Table 2 shows median scores at pre-test and post-test on each of the four scales used to assess the children’s clinical symptoms, as well as results of the Wilcoxon signed-rank test and effect sizes associated with differences between ranks.

The first point to note is that although scores on the CBCL indicated that the majority of children had clinical or sub-clinical levels of internalizing, externalizing and post-traumatic stress symptoms, 30% of the sample scored below the cut-offs for the presence of these psychological problems.

In order to examine the impact (post-test vs. pre-test) of the *Leaving a Mark* programme on the emotional and behavioural symptoms of children and adolescents who had been exposed to domestic violence we applied the Wilcoxon test. The results showed a statistically significant improvement in internalizing symptoms following participation in the programme (*Z* = −2.360; *p* = 0.018). The effect size associated with the difference between ranks (post-test vs. pre-test) was moderate (*r* = 0.38).

Regarding externalizing symptoms, post-test scores were not significantly better than those at pre-test (*Z* = −1.142; *p* = 0.253) and the effect size associated with the difference between ranks was small (*r* = 0.19). Thus, participation in the programme was not associated with a significant improvement in aggressive behaviour and rule-breaking behaviour.

By contrast, the results indicated that participation in the programme was associated with a statistically significant improvement in post-traumatic stress symptoms (*Z* = −2.407; *p* = 0.016). The effect size associated with the difference between ranks was moderate (*r* = 0.43).

Finally, although scores on the dysregulation profile (CBCL-DP) were lower at post-test, the difference was not statistically significant (*Z* = −1.656; *p* = 0.098) and the effect size associated with the difference between ranks was small (*r* = 0.27).

We then conducted a second analysis using the Mann-Whitney U test to examine whether there were differences between boys and girls both before and after their participation in the programme. The results are shown in Table 3.

It can be seen that at the pre-test, there was a statistically significant difference between boys and girls in externalizing symptoms and post-traumatic stress symptoms. The effect size associated with both comparisons was moderate. Although the difference between boys and girls on the other two pre-test measures was not statistically significant, the associated effect sizes were moderate. Therefore, and based on the recommendation to consider effect sizes when using non-parametric tests with small samples [57], we conclude that boys in general had more clinical symptoms than girls prior to participation in the AAI programme.

At the post-test, there was a statistically significant difference in the mean rank between boys and girls on all the clinical measures, with the exception of internalizing symptoms. The effect sizes associated with these comparisons were either moderate or large. These results indicate that after participation in the AAI programme, boys continued to present more behavioural and emotional problems than did girls.

## 4. Discussion

The aim of this study was to examine the impact of a pilot AAI programme, *Leaving a Mark*, on the clinical symptoms of children and adolescents who had been exposed to situations of domestic violence.

Although scores on the CBCL indicated that the majority of participants had clinical-level psychological problems, 30% of children were below the cut-off for clinical symptoms. This is consistent with previous studies that have reported an absence of emotional and behavioural problems in some children who have experienced traumatic events [20], a finding that has been linked to greater resilience [19].

Regarding internalizing and externalizing symptoms, the results showed that while participation in the programme was associated with a reduction in the former, there was no significant change in the latter. This suggests that the programme, in its current form, has a greater impact on internalizing as opposed to externalizing behaviours. The same finding was reported with a similar AAI programme aimed at adolescents who had experienced childhood trauma [30]. Consequently, a task for future research would be to determine if the improvement in internalizing —but not externalizing—symptoms is due specifically to the content of the *Leaving a Mark* programme or whether AAI programmes in general are better suited to addressing internalizing behaviour.

As we noted in the introduction to this paper, another common characteristic of children who have been exposed to domestic violence is the presence of symptoms associated with post-traumatic stress disorders [20]. In this respect, the results suggest that participation in the *Leaving a Mark* programme led to a reduction in symptoms of this kind (as measured by the CBCL-PTSP). This is a promising preliminary finding, since one of the primary aims of the programme is to reduce clinical symptoms associated with post-traumatic stress disorder. Bearing in mind the low dropout rate associated with AAI programmes in general, we consider that this result supports the use of the *Leaving a Mark* programme with children and adolescents who have been exposed to domestic violence.

The final area of clinical symptoms that we considered concerned an aspect that has scarcely been investigated in children exposed to gender-based violence, namely the CBCL Dysregulation Profile (CBCL-DP). This is an important aspect to explore, however, since elevated CBCL-DP scores in childhood have been reported to be predictive of severe mental health problems in adolescence and adulthood [46,48,49] and such problems are known to be common among individuals who have experienced childhood trauma. Consequently, in order to be considered effective, an intervention programme for children exposed to traumatic experiences such as domestic violence should lead to an improvement in the CBCL-DP. Contrary to our expectations, however, participation in the *Leaving a Mark* programme was not associated with such an improvement. Although this result needs to be verified in a larger sample, it nonetheless suggests that in order to effectively address affective and behavioural dysregulation, the *Leaving a Mark* programme would need to be complemented with a more specific approach, such as trauma-focused cognitive-behavioural therapy [55].

With respect to gender differences, and in line with meta-analytic findings [20,23], boys had more externalizing symptoms than girls at the pre-test, and they also scored higher on the measures of post-traumatic stress symptoms (CBCL-PTSP) and affective and behavioural dysregulation (CBCL-DP). These differences between boys and girls were maintained at the post-test. Thus, although participation in the *Leaving a Mark* programme tended to have a positive impact on the clinical symptoms of children and adolescents, boys continued to score higher than girls after the intervention. From a practical point of view, and although our results are preliminary and should be treated with caution, this suggests that it might be advisable to incorporate into the treatment programme a number of sessions specifically for boys, aimed at reducing their externalizing and post-traumatic stress symptoms.

The present study has a number of limitations. As it was a pilot study with a small sample, statistical power is low and the results must be interpreted with caution and should not be treated as generalizable. This is especially the case for the comparisons between boys and girls, since there were only six girls. Replication with a sufficiently large and representative sample is required before making inferences about the population of children exposed to domestic violence. In addition, and in order to isolate treatment effects from those attributable to other variables, future studies should include a matched control group of individuals who do not participate in the *Leaving a Mark* programme [58]. It would also be advisable to use multi-informant measures for the study variables of interest. For example, one could complement the CBCL data with appraisals by teachers and self-reports by children and adolescents themselves. Finally, the wide age range of our sample is another potential limitation of this pilot study. In future studies, with larger samples, it would be interesting to examine the impact of the intervention programme in different age groups (e.g. 6–8, 8–12 and 12–15).

## 5. Conclusions

The preliminary results obtained in this study suggest that the *Leaving a Mark* animal-assisted intervention programme may be effective in reducing at least some of the clinical symptoms presented by children and adolescents who have been exposed to domestic violence. The therapeutic benefits of working with animals, coupled with the low dropout rates associated with interventions of this kind, make such programmes an attractive alternative to traditional face-to-face approaches.

## Figures and Tables

**Table 1:** Summary of the *Leaving a Mark* programme.

Module	Session	Participants	Objectives
1. Establishing a secure base for the intervention	1	Child/adolescent	Establishing the therapeutic setting. Building the therapeutic alliance. Establishing a secure base.
	2	Child/adolescent	
2. Psychoeducation	3	Child/adolescent	Psychoeducation about exposure to gender-based violence and its emotional consequences.
	4	Child/adolescent	
	5	Child/adolescent + mother	
3. Arousal regulation and stress management	6	Child/adolescent	Promoting arousal regulation skills and strategies for coping with stress.
	7	Child/adolescent + mother	
4. Emotion expression and regulation	8	Child/adolescent	Encouraging emotional expression and the use of adaptive emotion regulation skills.
	9	Child/adolescent	
	10	Child/adolescent	
	11	Child/adolescent	
	12	Child/adolescent + mother	
5. Conclusion	13	Child/adolescent + mother	Bringing the intervention to a close and farewell.
	14	Child/adolescent	

**Table 2:** Median scores at pre-test and post-test on the scales used to assess clinical symptoms, results of the Wilcoxon signed-rank test and effect sizes associated with differences between ranks.

Variable	Assessment	Mdn.	N	Z	Sig.	Effect Size
Internalizing Problems (CBCL)	Pre-test	15.00	19	−2.360	0.018	0.38
	Post-test	12.00	19			
Externalizing Problems (CBCL)	Pre-test	16.00	19	−1.142	0.253	0.19
	Post-test	17.00	19			
CBCL-PTSP	Pre-test	12.00	19	−2.407	0.016	0.43
	Post-test	9.00	19			
CBCL-DP	Pre-test	30.00	19	−1.656	0.098	0.27
	Post-test	28.00	19			

**Table 3:** Mean ranks for boys (*n* = 13) and girls (*n* = 6) separately, results of the Mann-Whitney U test and effect sizes associated with differences between ranks.

Variables	Assessment	Boys		Girls		Mann-Whitney *U*			Effect Size
		Mean Rank	*N*	Mean Rank	N	U	Z	Sig.	*r*
Internalizing Symptoms (CBCL)	Pre-test	11.42	13	6.92	6	20.5	−1.627	0.104	0.37
	Post-test	11.31	13	7.17	6	22	−1.494	0.135	0.34
Externalizing Symptoms (CBCL)	Pre-test	11.81	13	6.08	6	15.5	−2.063	0.039	0.47
	Post-test	11.81	13	6.08	6	15.5	−2.063	0.039	0.47
CBCL-PTSP	Pre-test	11.73	13	6.25	6	16.5	−1.979	0.048	0.45
	Post-test	11.96	13	5.75	6	13.5	−2.253	0.024	0.52
CBCL-DP	Pre-test	11.69	13	6.33	6	17	−1.930	0.054	0.44
	Post-test	11.77	13	6.17	6	16	−2.018	0.044	0.46

## References

[1] United Nations (1993). The Declaration on the Elimination of Violence Against Women.

[2] World Health Organization (2019). Respect Women: Preventing Violence against Women.

[3] Coker A.L., Davis K.E., Arias I., Desai S., Sanderson M., Brandt H.M., Smith P.H. (2002). Physical and mental health effects of intimate partner violence for men and women. Am. J. Prev. Med..

[4] Hill A., Pallitto C., McCleary-Sills J., Garcia-Moreno C.A. (2016). Systematic review and meta-analysis of intimate partner violence during pregnancy and selected birth outcomes. Int. J. Gynecol. Obstet..

[5] World Health Organization (2013). Global and Regional Estimates of Violence against Women: Prevalence and Health Effects of Intimate Partner Violence and Non-Partner Sexual Violence.

[6] Tjaden P.G., Thoennes N. (2000). Full Report of the Prevalence, Incidence, and Consequences of Violence against Women.

[7] Li Y., Marshall C.M., Rees H.C., Nunez A., Ezeanolue E.E., Ehiri J.E. (2014). Intimate partner violence and HIV infection among women: A systematic review and meta-analysis. J. Int. AIDS Soc..

[8] Hall M., Chappell L.C., Parnell B.L., Seed P.T., Bewley S. (2014). Associations between intimate partner violence and termination of pregnancy: A systematic review and meta-analysis. PLoS Med..

[9] Campbell J.C. (2002). Health consequences of intimate partner violence. Lancet.

[10] Golding J.M. (1999). Intimate partner violence as a risk factor for mental disorders: A meta-analysis. J. Fam. Violence.

[11] Devries K.M., Child J.C., Bacchus L.J., Mak J., Falder G., Graham K., Heise L. (2014). Intimate partner violence victimization and alcohol consumption in women: A systematic review and meta-analysis. Addiction.

[12] Devries K.M., Mak J.Y., Bacchus L.J., Child J.C., Falder G., Petzold M., Watts C.H. (2013). Intimate partner violence and incident depressive symptoms and suicide attempts: A systematic review of longitudinal studies. PLoS Med..

[13] Holt S., Buckley H., Whelan S. (2008). The impact of exposure to domestic violence on children and young people: A review of the literature. Child Abuse Negl..

[14] Unicef (United Nations Children’s Fund) (2017). A Familiar Face: Violence in the Lives of Children and Adolescents.

[15] Osofsky J.D. (2003). Prevalence of children’s exposure to domestic violence and child maltreatment: Implications for prevention and intervention. Clin. Child Fam. Psychol. Rev..

[16] United Nations (2016). Global Strategy for Women’s, Children’s and Adolescents’ Health (2016–2030).

[17] Osofsky J.D. (1995). The effect of exposure to violence on young children. Am. Psychol..

[18] Rizo C.F., Macy R.J., Ermentrout D.M., Johns N.B. (2011). A review of family interventions for intimate partner violence with a child focus or child component. Aggress. Violent Behav..

[19] Howell K.H. (2011). Resilience and psychopathology in children exposed to family violence. Aggress. Violent Behav..

[20] Evans S.E., Davies C., DiLillo D. (2008). Exposure to domestic violence: A meta-analysis of child and adolescent outcomes. Aggress. Violent Behav..

[21] Kitzmann K.M., Gaylord N.K., Holt A.R., Kenny E.D. (2003). Child witnesses to domestic violence: A meta-analytic review. J. Consult. Clin. Psychol..

[22] Vu N.L., Jouriles E.N., McDonald R., Rosenfield D. (2016). Children’s exposure to intimate partner violence: A meta-analysis of longitudinal associations with child adjustment problems. Clin. Psychol. Rev..

[23] Wolfe D.A., Crooks C.V., Lee V., McIntyre-Smith A., Jaffe P.G. (2003). The effects of children’s exposure to domestic violence: A meta-analysis and critique. Clin. Child Fam. Psychol. Rev..

[24] Bayarri Fernàndez E., Ezpeleta L., Granero R., de la Osa N., Domènech J.M. (2011). Degree of exposure to domestic violence, psychopathology, and functional impairment in children and adolescents. J. Interpers. Violence.

[25] Cafferky B., Mendez M., Anderson J.R., Stith S.M. (2016). Substance use and intimate partner violence: A meta-analytic review. Psychol. Violence.

[26] Roustit C., Renahy E., Guernec G., Lesieur S., Parizot I., Chauvin P. (2009). Exposure to interparental violence and psychosocial maladjustment in the adult life course: Advocacy for early prevention. J. Epidemiol. Community Health.

[27] Fine A.H. (2015). Handbook on Animal-Assisted Therapy: Foundations and Guidelines for Animal-Assisted Interventions.

[28] Jones M.G., Rice S.M., Cotton S.M. (2019). Incorporating animal-assisted therapy in mental health treatments for adolescents: A systematic review of canine assisted psychotherapy. PLoS ONE.

[29] Kendall E., Maujean A., Pepping C.A., Downes M., Lakhani A., Byrne J., Macfarlane K. (2015). A systematic review of the efficacy of equine-assisted interventions on psychological outcomes. Eur. J. Psychother. Counsell. Health.

[30] Muela A., Balluerka N., Amiano N., Caldentey M.A., Aliri J. (2017). Animal-assisted psychotherapy for young people with behavioural problems in residential care. Clin. Psychol. Psychother..

[31] Carr E.C., Wallace J.E., Pater R., Gross D.P. (2019). Evaluating the Relationship between Well-Being and Living with a Dog for People with Chronic Low Back Pain: A Feasibility Study. Int. J. Environ. Res. Public Health.

[32] Viau R., Arsenault-Lapierre G., Fecteau S., Champagne N., Walker C.D., Lupien S. (2010). Effect of service dogs on salivary cortisol secretion in autistic children. Psychoneuroendocrinology.

[33] Friedmann E., Katcher A., Thomas S.A., Lynch J.J., Messent P.R. (1983). Social interaction and blood pressure: Influence of animal companions. J. Nerv. Ment. Dis..

[34] Cole K.M., Gawlinski A., Steers N., Kotlerman J. (2007). Animal-assisted therapy in patients hospitalized with heart failure. J. Crit. Care.

[35] Beetz A., Uvnäs-Moberg K., Julius H., Kotrschal K. (2012). Psychosocial and psychophysiological effects of human-animal interactions: The possible role of oxytocin. Front. Psychol..

[36] Germain S.M., Wilkie K.D., Milbourne V.M., Theule J. (2018). Animal-assisted psychotherapy and trauma: A meta-analysis. Anthrozoös.

[37] Imel Z.E., Laska K., Jakupcak M., Simpson T.L. (2013). Meta-analysis of dropout in treatments for posttraumatic stress disorder. J. Consult. Clin. Psychol..

[38] O’Haire M.E., Guérin N.A., Kirkham A.C. (2015). Animal-assisted intervention for trauma: A systematic literature review. Front. Psychol..

[39] Tedeschi P., Jenkins M.A. (2019). Transforming Trauma: Resilience and Healing through Our Connections with Animals.

[40] Balluerka N., Muela A., Amiano N., Caldentey M.A. (2015). Promoting psychosocial adaptation of youths in residential care through animal-assisted psychotherapy. Child Abuse Negl..

[41] Balluerka N., Muela A., Amiano N., Caldentey M.A. (2014). Influence of animal-assisted therapy (AAT) on the attachment representations of youth in residential care. Child. Youth Serv. Rev..

[42] Aitken M., Battaglia M., Marino C., Mahendran N., Andrade B.F. (2019). Clinical utility of the CBCL Dysregulation Profile in children with disruptive behavior. J. Affect. Disord..

[43] Caporino N.E., Herres J., Kendall P.C., Wolk C.B. (2016). Dysregulation in youth with anxiety disorders: Relationship to acute and 7-to 19-year follow-up outcomes of cognitive–behavioral therapy. Child Psychiatry Hum. Dev..

[44] Ayer L., Althoff R., Ivanova M., Rettew D., Waxler E., Sulman J., Hudziak J. (2009). Child Behavior Checklist Juvenile Bipolar Disorder (CBCL-JBD) and CBCL Posttraumatic Stress Problems (CBCL-PTSP) scales are measures of a single dysregulatory syndrome. J. Child Psychol. Psychiatry.

[45] Althoff R.R., Rettew D.C., Faraone S.V., Boomsma D.I., Hudziak J.J. (2006). Latent class analysis shows strong heritability of the child behavior checklist-juvenile bipolar phenotype. Biol. Psychiatry.

[46] Holtmann M., Buchmann A.F., Esser G., Schmidt M.H., Banaschewski T., Laucht M. (2011). The Child Behavior Checklist-Dysregulation Profile predicts substance use, suicidality, and functional impairment: A longitudinal analysis. J. Child Psychol. Psychiatry.

[47] Volk H.E., Todd R.D. (2007). Does the Child Behavior Checklist juvenile bipolar disorder phenotype identify bipolar disorder?. Biol. Psychiatry.

[48] De Caluwé E., Decuyper M., De Clercq B. (2013). The child behavior checklist dysregulation profile predicts adolescent DSM-5 pathological personality traits 4 years later. Eur. Child Adolesc. Psychiatry.

[49] Deutz M.H., Geeraerts S.B., van Baar A.L., Deković M., Prinzie P. (2016). The Dysregulation Profile in middle childhood and adolescence across reporters: Factor structure, measurement invariance, and links with self-harm and suicidal ideation. Eur. Child Adolesc. Psychiatry.

[50] Achenbach T.M., Rescorla L.A. (2001). Manual for ASEBA School-Age Forms & Profiles.

[51] Wolfe V.V., Gentile C., Wolfe D.A. (1989). The impact of sexual abuse on children: A PTSD formulation. Behav. Ther..

[52] Ivanova M.Y., Achenbach T.M., Rescorla L.A., Guo J., Althoff R.R., Kan K.J., da Rocha M.M. (2019). Testing syndromes of psychopathology in parent and youth ratings across societies. J. Clin. Child Adolesc. Psychiatry.

[53] Rescorla L.A., Althoff R.R., Ivanova M.Y., Achenbach T.M. (2019). Effects of society and culture on parents’ ratings of children’s mental health problems in 45 societies. Eur. Child Adolesc. Psychiatry.

[54] Sardinero E., Pedreira J.L., Muñiz J. (1997). El cuestionario CBCL de Achenbach: Adaptación española y aplicaciones clínico–epidemiológicas. Clin. Salud.

[55] Cohen J.A., Mannarino A.P., Deblinger E. (2017). Treating Trauma and Traumatic Grief in Children and Adolescents.

[56] Koeppen A.S. (1974). Relaxation training for children. Elem. Sch. Guid. Couns..

[57] Fritz C.O., Morris P.E., Richler J.J. (2012). Effect size estimates: Current use, calculations, and interpretation. J. Exp. Psychol. Gen..

[58] Comer J.S., Kendall P.C. (2013). The Oxford Handbook of Research Strategies for Clinical Psychology.

